# Integrated care initiatives in the Spanish Health System/Experiencias de integración asistencial en el Sistema Nacional de Salud de España: Abstracts from the Third Spanish Conference on Chronic Care, San Sebastián, 19–20 May 2011/Resúmenes de Comunicaciones al III Congreso Nacional de Atención Sanitaria al Paciente Crónico, Donostia-San Sebastián, 19 y 20 de mayo del 2011

**Published:** 2012-05-29

**Authors:** Roberto Nuño, Regina Sauto, Nuria Toro

**Affiliations:** O+Berri, Basque Institute for Healthcare Innovation, Spain; O+Berri, Basque Institute for Healthcare Innovation, Spain; O+Berri, Basque Institute for Healthcare Innovation, Spain

In developed countries, the evolution of medicine, technology and social protection has led to the development of complex health systems with multiple specialised healthcare providers, often working as separate islands. The Spanish health system is no exception.

There is a consensus that fragmentation of healthcare services particularly hampers the quality of care provided to chronic patients, who often require care from different types of professionals and specialists, as well as a long-term relationship with the healthcare system. Indeed, it can be argued that integrated care is particularly relevant to chronic patients and those with multiple conditions, who “bear the brunt of access, continuity, fragmentation and quality problems found in all health systems” [1]. With the growing epidemic of chronic diseases, which already represent the main burden of disease and are responsible for most interactions with healthcare providers in developed countries [2–5], improving the quality, effectiveness and efficiency of the care provided to chronic patients has become one of the major challenges for health systems.

In this context, the annual Spanish Conference on Chronic Care grew out of an interest in sharing experiences, knowledge and views on innovative approaches and initiatives in the prevention and management of chronic diseases, among healthcare experts, managers, clinicians and policy makers in the field. As in previous years, during the Third Spanish Conference on Chronic Care, “continuity of care” and “integrated care” were recurring topics, and recognised as key for the improvement of care for chronic patients. On this occasion, the conference, held in San Sebastian in May 2011, was organised by the Basque Institute for Healthcare Innovation (O+Berri) and the Basque Health Service (Osakidetza), with the support of the Department of Health and Consumer Affairs of the Basque Government. The present supplement includes a selection of 19 abstracts chosen from among the oral presentations and posters on interventions, projects and studies on healthcare coordination and integrated care presented at the conference.

Fragmentation of healthcare delivery is commonplace in the Spanish health system, despite centralised public ownership of healthcare services in most regions. In Spain, the organisation of the healthcare system is devolved to the autonomous regions, which has led to the development of 17 separate regional health services [6]. Financed through taxes, regional health services in Spain provide primary and specialised care, free of charge (except for outpatient prescriptions, for which there is copayment) to all residents. Each person has an assigned general practitioner, who acts as a gatekeeper to the rest of the system. Healthcare providers at all levels of care are predominantly public, and most health professionals are employees with civil servant status. In general, public healthcare providers in each region are owned by a public organisation, which centrally oversees the regional health service. This does not, however, prevent the Spanish system from suffering problems in the coordination and integration of healthcare between different care levels.

In addition, the coordination of healthcare and social care, which are often both required for chronic patients, especially among the elderly, is particularly hindered by the diversity of institutions involved. In most Spanish regions, long term and social care for the elderly and disabled falls outside the remit of the health authorities, making its coordination and integration with health care even more difficult.

The sample of initiatives covered in this conference supplement illustrates how the concept of “integrated care” is being implemented by practitioners in Spain. It includes examples from different regional health systems: the Basque Country (7 abstracts), Catalonia (6), Valencia (2), Andalusia (1), the Canary Islands (1), and Castile—La Mancha (1), as well as a study with participants from twelve autonomous regions (the Esperanza Project). With this publication, we wish to contribute a Spanish perspective on the development of the multifaceted and dynamic concepts of “integrated care”, “healthcare integration”, and “continuity of care”, among others [7–9], as well as the solutions being proposed to achieve their associated goals.

Different models of integration are emerging across Spain [10, 11]. Within this trend, two main approaches can be distinguished: one that focuses on clinical processes (micro level), and another that is mostly concerned with the organisational architecture of the health delivery system (meso to macro level). Models for clinical integration usually focus on one or several healthcare processes, and are the most common. Within these models, the care processes for patients with multiple pathologies have received particular attention and their use is becoming fairly widespread [12, 13]. A second approach to integration, which coexists with clinical integration initiatives, is the creation of integrated health organisations that join together, under a single legal entity, separate providers of primary and hospital care. Such integrated health organisations, which are in line with Shortell et al.’s proposed model [14], are being created in several regions in Spain. They share some common elements: patient-centeredness, a systemic perspective, a population management approach, an emphasis on coordination of services, and a focus on outcomes [8, 15]. This publication includes examples of initiatives from several of these organisations: the Bidasoa Integrated Health Organisation (Berraondo), the Bizkaia Mental Health Services (Pereira Rodriguez et al.), the Baix Empordà Integrated Health Service (Pérez Berruezo et al.), and the Integrated Health Consortium of Hospitalet (Sánchez Chamero et al.).

All the interventions described in the abstracts of this conference supplement take a clinical approach to integration, focussing on one or several care processes, even if some are run within integrated health organisations. On the other hand, some focus on care processes for patients with multiple chronic conditions, while others follow a disease management approach.

## Management of patients with multiple chronic diseases

For this group of patients, which would correspond to those at the top of the Kaiser Permanente pyramid [16], the benefits of integrated care and of a proactive follow-up from the healthcare providers seem particularly evident. Indeed, the current organisation of healthcare delivery is especially inappropriate for these patients, who account for the highest share of preventable hospitalisations and are responsible for most healthcare expenditure [17]. Nevertheless, there are still huge gaps in our knowledge concerning which therapies and care strategies work best for comorbid patients. For example, such individuals are often excluded from clinical trials, which have traditionally focused on patients with a single disease [18]. In the abstracts presented here, patients with multiple chronic diseases are also referred to as complex patients, frail patients, patients with repeated admissions to hospital, or high-risk high-cost patients (Artetxe et al., Vallejo Maroto et al., Pérez Berruezo et al., Francisco Lucena et al., Vercher González et al., Machín Lázaro et al., Gallud et al., García Cerdán et al., and Berraondo).

The interventions presented illustrate the following trends towards integrated care for patients with multiple chronic conditions or complex patients in Spain:

- Units, teams or specialists with a remit for multimorbid patients at the hospital are being developed, often within units of internal medicine. The assigned internal medicine physician and/or continuity of care unit act as a consultant to the primary care level and also ensure a stable point of contact for the patient at the hospital [13].
- Case managers are being used, a role most often given to nurses.
- The improvement of integrated care for complex patients is, in most cases, carried out within broader initiatives for the management of chronic patients. Consistent with the Chronic Care Model and related frameworks [16, 19], these initiatives tend to include not only changes in the organisation of healthcare delivery, but also some of the following elements: education for patients and caregivers, individual care plans, access to shared electronic medical records by different care providers, the development of proactive relationships with patients, and remote communication technologies, among others.
- Interventions are being evaluated in terms of their impact on the use of healthcare resources, and particularly on: number of hospitalisations, length of hospital stay, readmissions, and number of emergency department attendances.
- A high proportion of the complex patients targeted have a diagnosis of heart failure and/or chronic obstructive pulmonary disease.

These abstracts underline the importance of identifying the group of complex patients to be targeted by a given intervention. A range of different population stratification methods and criteria (Ollero et al.’s categories [12], ACGs, CRGs, among others) are currently being tested and used. The initiatives discussed also reflect the fact that ensuring continuity of care for complex patients, often characterised by old age and/or a high degree of functional dependence, requires healthcare to be coordinated with long-term care and social services. As noted above, this is a special challenge in the Spanish system, where these areas are often not the responsibility of the same public authorities, or branches of public administration, as healthcare. Another conclusion that can be derived from several of the interventions reported here is that when designing and implementing interventions for complex patients, the key role of the “informal caregiver” needs to be taken into consideration.

## Disease management

A second group of abstracts (Pérez Cánovas et al., Alvarez de Arcaya Vitoria et al., Marichal Planas et al., Sans Corrales et al., Carmona, Pereira Rodriguez et al., Sánchez Chamero et al., and Merino Hernandez et al.) concern disease management and related approaches. These include examples of:

- Virtual consultations between a hospital endocrinology service and primary care doctors
- Agreed protocols on the care and referral of patients with hip fracture between an acute and a rehabilitation hospital
- A shared disease management programme between primary care and an acute hospital for patients with heart failure
- Development of shared care pathways between primary care and specialised care for chronic obstructive pulmonary disease, heart failure, diabetes mellitus type 2 and spondyloarthritis
- Assertive community treatment for patients with severe mental disorders
- A study of variability in practice for depression by care setting (primary care versus specialised care)
- The management of an initiative that combines and coordinates several interventions for clinical integration in a health region.

The diversity of initiatives and approaches under way highlights that there are neither magical recipes nor definitive evidence about what works best in integrated care. On the other hand, there are plenty of examples showing that business as usual does not work for chronic patients and that there is huge room for improvement [20]. Integration has been found to be positively correlated with the presence of elements of the Chronic Care Model, a model guiding change towards improved chronic care [21]. Indeed, several reviews have reported that improved care coordination and integration have a positive impact on both processes and outcomes of care [19, 22, 23]. In several cases, interventions towards integration have also been found to be cost-effective, especially when an effort is made to adequately identify and reach patients most at risk of health deterioration [23]. However, a holistic framework addressing the multidimensionality of integrated care is still lacking. In relation to this, the last abstract included in this supplement (Toro et al.), proposes a multidimensional framework for assessing integration processes at a health system level, which can be adapted to different local contexts. This evaluation framework includes, among its indicators of integrated care outcomes, measures of the satisfaction of patients and professionals, accessibility, clinical effectiveness, quality of life and efficiency. Other tools for evaluating health system improvement towards better chronic care developed in Spain, such as the ARCHO (Assessment of Readiness for Chronicity in Health Care Organizations) [24], were also presented at the conference.

The projects and initiatives included in this conference supplement add to the body of evidence on integration strategies for improving care for chronic patients in different contexts.

## Editorial Español

En los países avanzados, el progreso de la medicina, la tecnología y los sistemas de protección social, ha llevado al desarrollo de complejos sistemas sanitarios en los que intervienen múltiples proveedores de servicios sanitarios, que a menudo funcionan de forma aislada. El caso del sistema nacional de salud español no es una excepción.

Existe amplio consenso sobre el efecto perjudicial de la fragmentación de los servicios sanitarios sobre la calidad de la atención, especialmente a los pacientes crónicos, ya que estos habitualmente requieren asistencia por parte de distintos tipos de profesionales, así como una relación a largo plazo con el sistema sanitario. Por ello, puede decirse que una atención integrada es especialmente relevante para los pacientes crónicos y pluripatológicos, que son los que más sufren los problemas de acceso, continuidad, fragmentación y calidad, presentes en todos los sistemas sanitarios [1]. Además, la epidemia de enfermedades crónicas, que representan la mayor carga de morbi-mortalidad en los países desarrollados [2–5], ha convertido la mejora de la calidad, efectividad y eficiencia de la atención a los pacientes crónicos en uno de los mayores retos para los sistemas sanitarios en estos países.

El Congreso Nacional de Atención Sanitaria al Paciente Crónico (de periodicidad anual) surgió del interés de diversos expertos sanitarios, gestores, clínicos y decisores políticos en compartir experiencias, conocimientos y perspectivas, en cuanto a enfoques e iniciativas innovadoras en prevención y gestión de enfermedades crónicas. Como en ediciones anteriores, durante el III Congreso Nacional de Atención Sanitaria al Paciente Crónico, la “atención integrada y continuidad asistencial” ha sido un tema recurrente, considerándose un área clave para la mejora de la atención a las enfermedades crónicas. El citado congreso se celebró en Donostia-San Sebastián en mayo del 2011, y fue organizado por O+berri (Instituto Vasco de Innovación Sanitaria) y Osakidetza (Servicio Vasco de Salud), con el apoyo del Departamento de Sanidad y Consumo del Gobierno Vasco. Este suplemento congresual incluye una selección de 19 comunicaciones elegidas entre las ponencias, comunicaciones orales y pósters sobre experiencias, proyectos y estudios en coordinación e integración asistencial presentados durante el congreso.

Puede decirse que la fragmentación en la provisión de asistencia sanitaria es algo común en el sistema español de salud, a pesar de que en la mayoría de las Comunidades Autónomas (CCAA), los proveedores de servicios sanitarios sean de titularidad pública. En España, la competencia en cuanto a la organización de la asistencia sanitaria se encuentra a nivel regional, a cargo de las CCAAs, lo que ha conducido al desarrollo de 17 servicios regionales de salud diferenciados [6]. Financiados vía impuestos, los servicios regionales de salud proveen de atención primaria y especializada a todos los residentes de forma gratuita (excepto para fármacos dispensados en las oficinas de farmacia, para los que existe un sistema de copago). Cada residente tiene asignado un médico de atención primaria, que actúa de puerta de entrada al resto del sistema. Los proveedores sanitarios son predominantemente públicos, en todos los niveles de asistencia, y los profesionales sanitarios tienen en su mayoría status funcionarial. De forma general, y con algunas excepciones, las organizaciones sanitarias públicas de cada Comunidad Autónoma son propiedad de un ente público, que controla de forma corporativa el servicio regional de salud. Esto no impide, sin embargo, que el sistema español sufra de problemas de coordinación e integración entre distintos ámbitos asistenciales.

Asimismo, la coordinación entre la atención sanitaria y la socio-sanitaria, que a menudo también requieren los pacientes crónicos, sobre todo aquellos de edad más avanzada, se ve especialmente obstaculizada por la pluralidad de instituciones proveedoras involucradas. Además, en la mayoría de las CCAAs, la atención sanitaria por una parte y la atención socio-sanitaria y social a mayores y dependientes por otra, son competencia de distintas autoridades públicas, lo que dificulta aún más su coordinación e integración.

La muestra de experiencias recogidas en este suplemento pretende ilustrar la manera en la que el concepto de “atención integrada” se está empleando actualmente en España. Así, reúne ejemplos de distintos sistemas regionales de salud: País Vasco (7 casos), Cataluña (6), Valencia (2), Andalucía (1), Islas Canarias (1), y Castilla-La Mancha (1), así como un estudio en el que colaboran participantes de doce CCAAs (el proyecto Esperanza). Con esta publicación se espera contribuir, con una perspectiva española, al desarrollo de los conceptos multifacéticos y dinámicos de “atención integrada”, “integración asistencial”, y “continuidad asistencial”, entre otros [7–9], así como a la propuesta de estrategias para avanzar en su desarrollo.

Así, existen distintos modelos de integración asistencial que están emergiendo en España [10, 11], entre los que cabe distinguir dos enfoque principales: uno orientado hacia la integración de los procesos clínicos (a nivel micro), y otro que toma una perspectiva más estructural y se centra en la arquitectura organizacional del sistema de provisión sanitaria (a niveles meso y macro). Los modelos de integración clínica son los más extendidos, centrándose normalmente en uno o varios procesos clínicos. Dentro de estos modelos, los procesos de atención a los pacientes pluripatológicos han recibido una atención especial, y su uso está extendiéndose de forma destacable [12, 13]. Un segundo enfoque hacia la integración, que convive con las iniciativas de integración clínica, es la creación de Organizaciones Sanitarias Integradas (OSIs) que reúnen, en una misma organización, proveedores de atención primaria y hospitalaria, que hasta entonces funcionaban separadamente. Estas OSIs, asimilables, al menos parcialmente, al modelo propuesto por Shortell et al. [14], están siendo creadas en distintas CCAAs, y comparten algunos elementos comunes como: orientación al paciente, perspectiva sistémica, enfoque de gestión poblacional, énfasis en la coordinación de servicios, y orientación a resultados [8, 15]. Esta publicación incluye ejemplos de experiencias de varias de estas organizaciones: OSI Bidasoa (Berraondo), Red de Salud Mental de Bizkaia (Pereira Rodriguez et al.), *Serveis de Salut Integrats Baix Empordà* (Pérez Berruezo et al.), y el *Consorci Sanitari Integral—Hospital General d’Hospitalet* (Sánchez Chamero et al.).

Las iniciativas descritas en este suplemento congresual se refieren a experiencias de integración asistencial a nivel micro, que se centran en uno o varios procesos clínicos, incluso si algunas se desarrollan en el marco de OSIs. Entre ellas, algunas se centran en los procesos de los pacientes con múltiples patologías o complejos, y otras siguen un enfoque de gestión de enfermedades.

## Gestión de pacientes pluripatológicos

Para este grupo de pacientes, que correspondería a los que se encuentran en el vértice de la pirámide de Kaiser [16], los beneficios de la atención integrada y de un seguimiento proactivo por parte de los proveedores sanitarios, parece especialmente evidente. De hecho, la actual organización de la asistencia sanitaria resulta especialmente inapropiada para este tipo de pacientes, que sufren la mayor parte de las hospitalizaciones evitables y concentran la mayor parte del gasto sanitario [17]. Todavía existe, sin embargo, una importante laguna en el conocimiento de las terapias y estrategias asistenciales más adecuadas para los pacientes con comorbilidad o multimorbilidad, quienes, por ejemplo, son a menudo excluidos de los ensayos clínicos, tradicionalmente realizados en pacientes con una única patología [18]. En los resúmenes que aquí se incluyen, se usan distintos términos para referirse a estos pacientes pluripatológicos, como son: pacientes complejos, frágiles, reingresadores, o en riesgo de alto consumo de recursos (resúmenes de Artetxe et al., Vallejo Maroto et al., Pérez Berruezo et al., Francisco Lucena et al., Vercher González et al., Machín Lázaro et al., Gallud et al., García Cerdán et al., Berraondo).

En las experiencias dirigidas a la integración asistencial para pacientes pluripatológicos o complejos en España, cabe observar algunas tendencias como son:

- La creación o identificación de unidades, equipos o especialistas responsables de los pacientes pluripatológicos en el hospital, a menudo dentro de unidades de medicina interna. En estos modelos, el médico de medicina interna de referencia o la unidad de continuidad asistencial, según el caso, actúan como consultores para el nivel de atención primaria, y aseguran un punto de contacto estable para el paciente en el hospital [13].
- El uso de gestoras de caso, un rol normalmente desempeñado por profesionales de enfermería.
- La mejora de la integración asistencial en pacientes complejos a menudo se desarrolla en el marco de iniciativas de gestión de crónicos más amplias. Así, en línea con el *Chronic Care Model* y otros modelos relacionados [16, 19], estas iniciativas tienden a incluir cambios, no sólo en la organización de la provisión sanitaria, sino también en algunos otros de los siguientes elementos: educación a los pacientes y cuidadores, uso de planes individualizados de cuidados, acceso compartido a la historia clínica del paciente por parte de distintos proveedores, desarrollo de relaciones proactivas con los pacientes, y uso de tecnologías de comunicación a distancia, entre otros.
- Suele medirse el efecto de las intervenciones en el uso de recursos sanitarios, y especialmente en los siguientes indicadores: número de hospitalizaciones, estancia hospitalaria, reingresos y número de visitas a urgencias.
- Gran parte de los pacientes complejos objeto de estas intervenciones tienen diagnóstico de insuficiencia cardiaca o de enfermedad pulmonar obstructiva crónica (EPOC).

Además, estos resúmenes ponen de relieve la importancia de los métodos utilizados para identificar al grupo de pacientes complejos que va a ser objeto de una determinada intervención. Actualmente se están testando y utilizando distintos métodos y criterios de estratificación de la población (categorías de Ollero et al. [12], ACGs, CRGs, entre otros). Las experiencias presentadas reflejan también el hecho de que para asegurar la continuidad asistencial de los pacientes complejos, a menudo caracterizados por su edad avanzada y por un considerable grado de dependencia, es necesario coordinar la atención sanitaria con los servicios socio-sanitarios y sociales. Como se ha mencionado anteriormente, este es un desafío importante para el sistema español, en el que estas áreas son a menudo responsabilidad de distintas autoridades o departamentos de la administración pública. Otra conclusión que cabe derivar de las intervenciones recogidas en pacientes complejos, es el papel clave que juega el cuidador informal en la atención a este tipo de pacientes, y la importancia de considerarlo en el diseño e implantación de las intervenciones.

## Gestión de enfermedades

Un segundo grupo de experiencias en este suplemento trata sobre gestión de enfermedades y enfoques similares (Pérez Cánovas et al., Alvarez de Arcaya Vitoria et al., Marichal Planas et al., Sans Corrales et al., Carmona, Pereira Rodriguez et al., Sánchez Chamero et al., Merino Hernandez et al.). Estos incluyen ejemplos de:

- Consulta virtual entre el servicio de endocrinología de un hospital y médicos de atención primaria.
- Protocolos compartidos para la atención y derivación de pacientes con fractura de cadera entre un hospital agudo y un hospital de rehabilitación.
- Un programa de enfermedad compartido entre atención primaria y un hospital de agudos para pacientes con fallo cardiaco.
- Desarrollo de rutas asistenciales compartidas entre atención primaria y atención especializada para EPOC, fallo cardiaco, diabetes mellitus tipo 2 y espondiloartritis.
- Tratamiento asertivo comunitario para pacientes con trastorno mental grave.
- Un estudio sobre la variabilidad de la práctica clínica en depresión según el dispositivo asistencial (atención primaria frente a atención especializada).
- La gestión de una iniciativa que combina y coordina varias intervenciones de integración clínica en una comarca sanitaria.

La diversidad de las experiencias y enfoques existentes pone de manifiesto el hecho de que no hay recetas mágicas ni evidencia definitiva sobre qué funciona mejor en integración asistencial. Sin embargo, hay numerosos ejemplos de que la manera en la que actualmente se gestiona al paciente crónico no es la adecuada, y de que hay un amplio margen para la mejora en su atención [20]. Asimismo, la integración asistencial ha mostrado correlacionarse positivamente con los componentes del *Chronic Care Model*, modelo que sirve de hoja de ruta hacia una mejor atención a crónicos [21]. De hecho, varias revisiones han mostrado cómo la mejora de la coordinación e integración asistencial tiene un impacto positivo tanto en procesos como en resultados de la asistencia [19, 22, 23]. En varios casos, las intervenciones hacia la integración asistencial han mostrado ser también coste-efectivas, especialmente cuando se ha hecho un esfuerzo por identificar adecuadamente y alcanzar a los pacientes con mayor riesgo de deterioro en su estado de salud [23]. Aún falta, sin embargo, un marco holístico que contemple la multidimensionalidad de la atención integrada. En este sentido, el último resumen incluido en este suplemento (Toro et al.), propone un marco multidimensional para la evaluación de los procesos de integración a nivel de un sistema sanitario, que puede adaptarse a distintos contextos locales. Este modelo evaluativo incluye, entre sus indicadores de resultados de la asistencia integrada, medidas de satisfacción de los pacientes y profesionales, accesibilidad, efectividad clínica, calidad de vida y eficiencia. Además, en este congreso también se presentaron otras herramientas de evaluación del avance de los sistemas sanitarios hacia una mejor atención a los pacientes crónicos desarrolladas en España, como el IEMAC (Instrumento de Evaluación de Modelos de Atención ante la Cronicidad) [24].

Esperamos que los distintos proyectos y experiencias incluidos en este suplemento contribuyan a la evidencia sobre estrategias de integración para la mejora de la atención a pacientes crónicos en distintos contextos.
